# Supplementary material for: Cerebellar output shapes cortical preparatory activity during motor adaptation
Source: Nat Commun. 2025 Mar 15;16:2574. doi: 10.1038/s41467-025-57832-4 (PMC11910607; doi:10.1038/s41467-025-57832-4)
Supplement: Supplementary file 1 — Supplemental Information [file 41467_2025_57832_MOESM1_ESM.pdf]

Supplemental information

Cerebellar output shapes cortical preparatory activity during motor adaptation

Sharon Israely*[1], Hugo Ninou*[1,2,3], Ori Rajchert[4], Lee Elmaleh[1], Ran Harel[5], Firas Mawase[4], Jonathan Kadmon¥[1], Yifat Prut¥[1]

[1] The Edmond and Lily Safra Center For Brain Sciences, The Hebrew University, Jerusalem, 91904-01, Israel
[2] Laboratoire de Neurosciences Cognitives et Computationnelles, INSERM U960, Département D'Etudes Cognitives, Ecole Normale Supérieure, PSL University, Paris, France
[3] Laboratoire de Physique de l'Ecole Normale Superieure, Ecole Normale Supérieure, PSL University, Paris, France
[4] Faculty of Biomedical Engineering, Technion - Israel Institute of Technology, Haifa, Israel
[5] Department of Neurosurgery, Sheba Medical Center, 5262000 Tel Aviv, Israel

*These authors contributed equally.

¥Corresponding authors: yifat.prut@mail.huji.ac.il and jonathan.kadmon@mail.huji.ac.il

Table of content

Supplemental Figure S1	3
Supplemental Figure S2	3
Supplemental Figure S3	4
Supplemental Figure S4	5
Supplemental Figure S5	5
Supplemental Figure S6	6
Supplemental Figure S7	7
Supplemental Figure S8	7
Supplemental Table 1	8

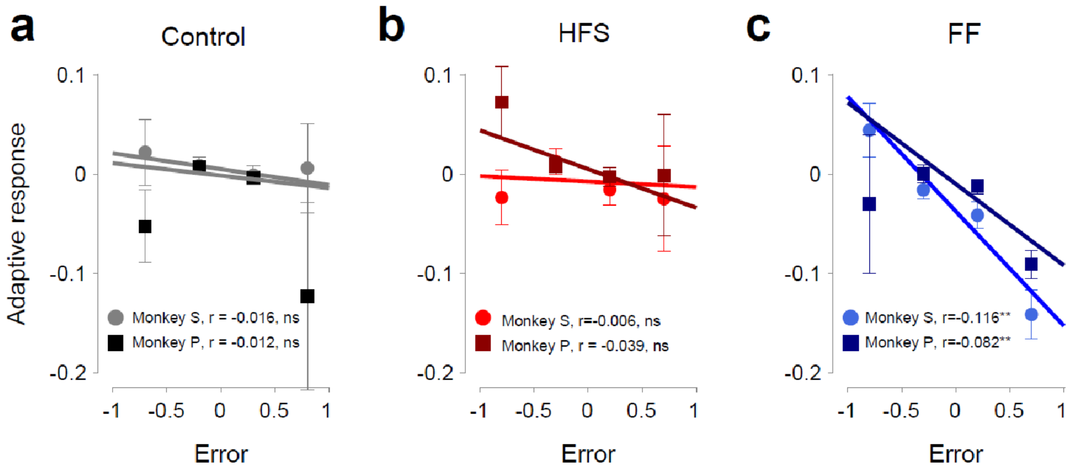

Supplementary Figure S1. Monkey-specific relations between adaptive response and error in different trial conditions. Monkey-specific adaptive responses were calculated for each monkey during the Control conditions (**a**), HFS (**b**) and FF (**c**). The regression slope is shown on each graph.

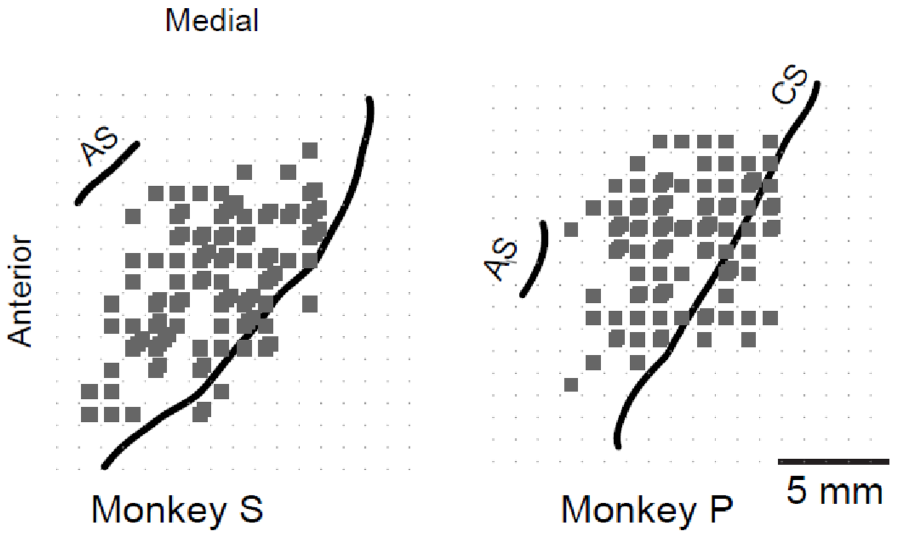

Supplementary Figure S2. Recording maps. Location of cortical recordings obtained in Monkey S (left) and Monkey P in relation to known landmarks. CS: central sulcus. AS: arcuate sulcus.

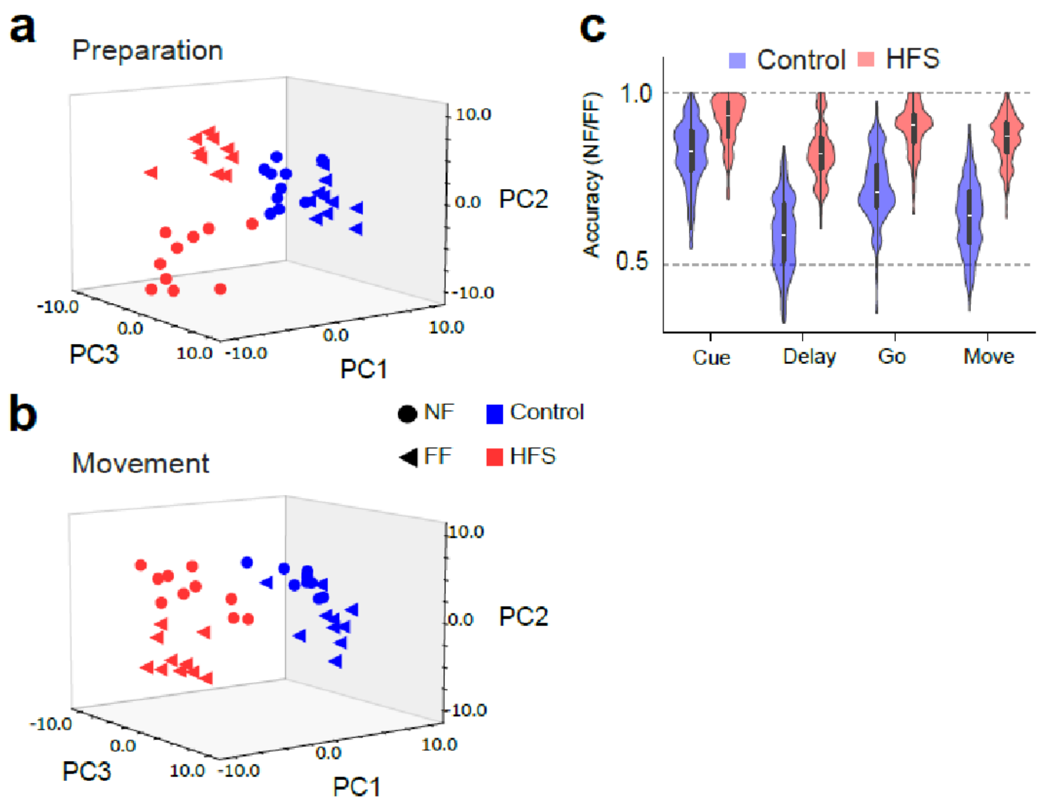

Supplementary Figure S3. Single-trial projection decoding accuracy calculated for Monkey P. Unlike Monkey S (Fig. 4a-c), here we only had 10 trials for the 4 different conditions. **a,** Projection of trial-specific coordinated activity on the first 3 PCs during preparation for movement. Each dot represents coordinated activity in a single trial during the sequence of trials performed in control (blue) or HFS (red) conditions, either before adaptation (null field, NF - circles) or during adaptation (FF - triangles). Neural data were averaged across all targets and obtained during a time window spanning -300 ms to 0 around the Go signal. Number of cells is n=94 for all figure panels. **b,** Same as a but calculated during the 100-400 ms after the Go signal. **c,** Decoding accuracy of adaptation conditions (NF vs. FF) based on epoch-specific data for the control (blue bars) or HFS (red bars). The dashed line denotes chance level (0.5). The different values for decoding accuracy were obtained for different realizations of the training and testing sets.

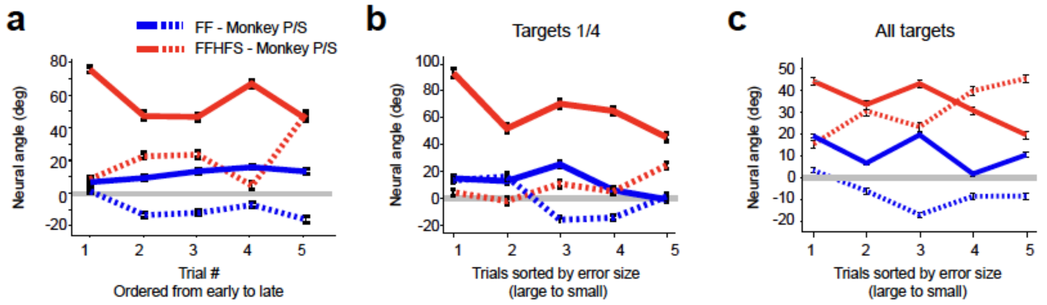

Supplementary Figure S4. Adaptation-related changed in the neural angle. a, Average neural angle as a function of the trial number, ordered from early trials (with high maximal deviation values) to late trials (with low maximal deviations). Neural angles for monkeys P and S are shown respectively in full and dashed lines. Blue and red, respectively indicate the FF and FF-HFS conditions. For monkey P we only used adaptation sessions with learned targets that required inter-joint coordination (4 and 8). Number of cells used for this analysis is n=241 for monkey P and n=378 for monkey S. The same number of cells was used for all figure panels. **b,** Neural angle calculated for both monkeys only using adaptation sessions with learned targets that required inter-joint coordination (4 and 8). Trials are ordered by error size from large to small errors. **c**, Same as b but taking all targets for both monkeys.

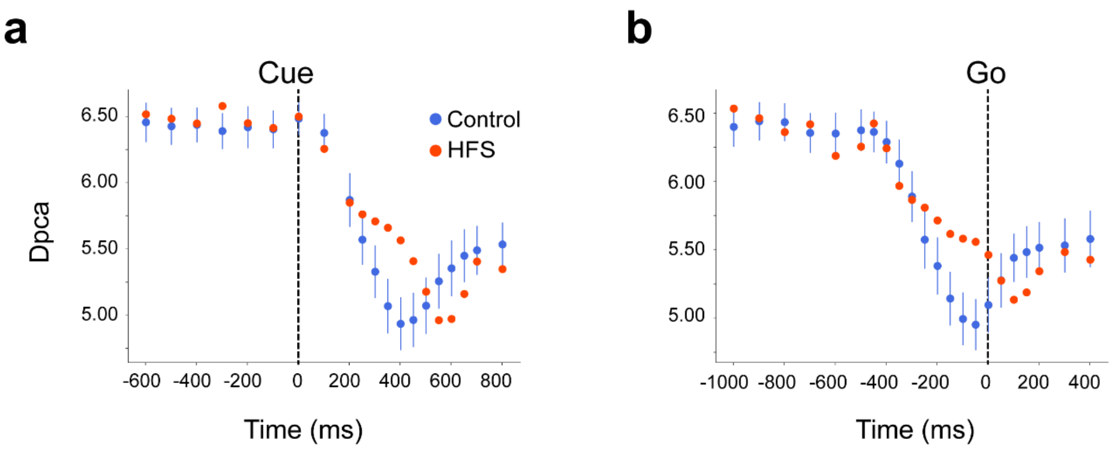

Supplementary Figure S5. Time-resolved modulation of participation ratio (Dpca). The Dpca was calculated (see Methods) in time bins of 5 ms, sampled every 100 ms. Calculations were performed on data aligned on the Cue (**a**) or the Go (**b**) events. Since in almost all cases there were more trials available in the Control than in the HFS conditions, we used a resampling method on the control data to obtain confidence intervals, shown as bars around control data (corresponding to the standard deviations). Number of cells is n=74.

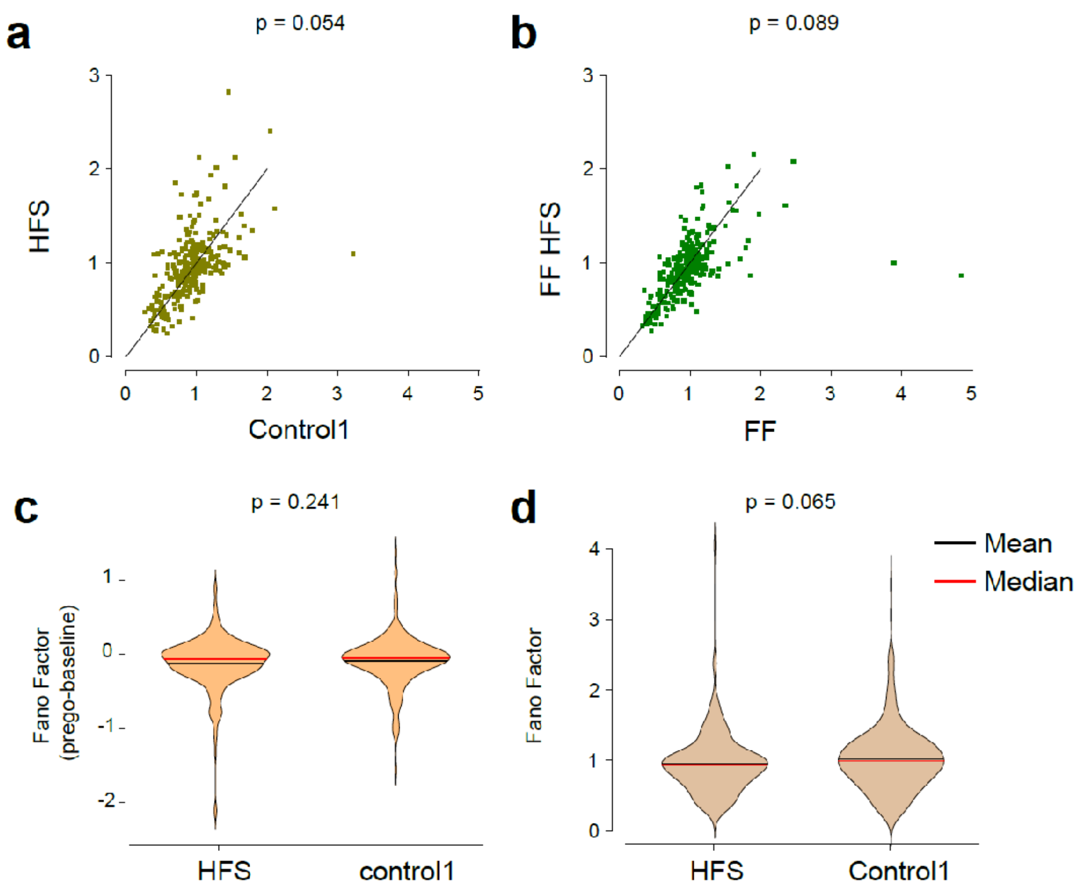

Supplementary Figure S6. Effect of HFS on trial-trial variability. We used the Fano factor to estimate trial-to-trial neural variability. The pre-go Fano factor was calculated around the Go signal (-300 ms to + 100 ms) when the monkeys made repeated movements to the Learned Target. **a,** pairwise comparison of Fano factors obtained for single cells in the control (x-axis) and HFS (y-axis) conditions. **b**, Same as a but for the FF (x-axis) and FF-HFS (y-axis) conditions. In both cases a paired t-test revealed no significant differences. **c**, Population-based comparison of mean changes in Fano factor (reduction in the Fano factor during the pre-Go vs. pre-Cue levels) calculated during the HFS and Control conditions. **d,** same as c but comparing the non-subtracted Fano factor. No significant differences were found in either case.

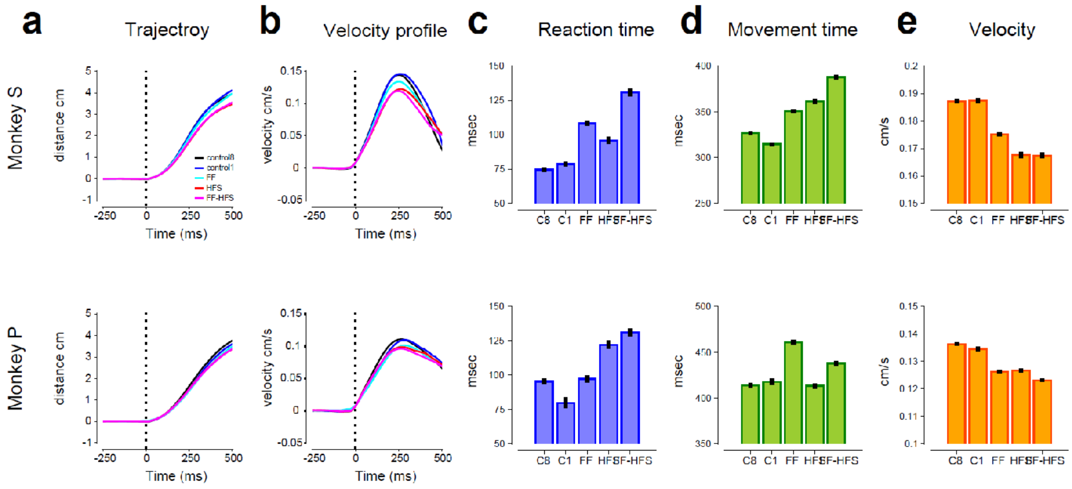

Supplementary Figure S7. Analysis of behavioral parameters. The behavioral properties and the effect of HFS were analyzed for each monkey (top row - monkey S, bottom row - monkey P). We calculated the movement trajectories in the different conditions and velocity profiles. We also measured the mean (and SEM) for the reaction time, movement time and peak velocities in each task condition. C8 corresponds to the Control condition in which the monkeys acquired 1 of 8 targets. C1 corresponds to the Control conditions in which the monkeys made a movement towards the one and only target (the Learned Target - LT). In all the remaining conditions the monkeys only made movements to the LT. Results of statistical analyses (2-way analysis of variance) are shown in Supplemental Table 1.

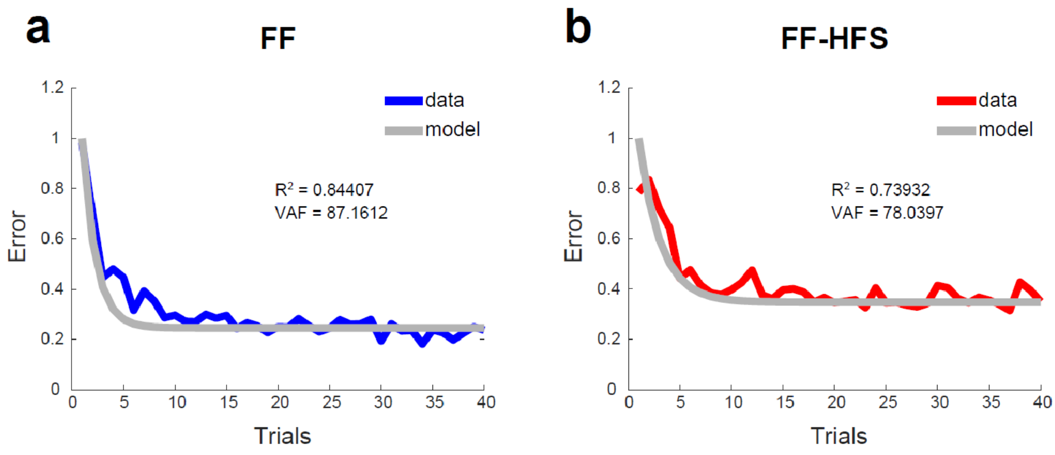

Supplementary Figure S8. Condition-specific goodness of fit. Calculated retention factor and error sensitivity were used to compare the model to the data (median of trial-dependent error calculated for the learning sessions) to test the R-squared and variance Accounted For in the FF (**a**) and FF-HFS (**b**) conditions.

		RT			MT			Peak Vel		
**Monkey**	**Source**	**DF**	**F**	**P**	**DF**	**F**	**P**	**DF**	**F**	**P**
P	FF	1	18.84	<< 0.001	1	139.22	<< 0.001	1	65.42	<< 0.001
P	HFS	1	158.76	<< 0.001	1	22.78	<< 0.001	1	57.78	<< 0.001
P	FF*HFS	1	1.97	0.16 ns	1	10.71	0.0011	1	10.51	0.0012
S	FF	1	203.91	<< 0.001	1	198.43	<< 0.001	1	46.72	<< 0.001
S	HFS	1	75.55	<< 0.001	1	361.17	<< 0.001	1	227.91	<< 0.001
S	FF*HFS	1	1.33	0.25 ns	1	4.81	0.028	1	44.12	<< 0.001

Supplemental Table 1. Monkey-specific results of 2-way ANOVAs measuring the effect of FF, HFS and their interactions on reaction time (RT), Movement time (MT) and Peak velocity (Peak Vel)
